# Identifying Vulnerable Populations Using a Social Determinants of Health Framework: Analysis of National Survey Data across Six Asia-Pacific Countries

**DOI:** 10.1371/journal.pone.0083000

**Published:** 2013-12-09

**Authors:** Paul R. Ward, Loreen Mamerow, Samantha B. Meyer

**Affiliations:** 1 Discipline of Public Health, Flinders University, Adelaide, South Australia, Australia; 2 School of Public Health and Health Systems, University of Waterloo, Waterloo, Ontario, Canada; CUNY, United States of America

## Abstract

**Background:**

In order to improve the health of the most vulnerable groups in society, the WHO called for research on the multiple and inter-linking factors shaping the social determinants of health (SDH). This paper analyses four key SDH (social cohesion, social inclusion, social empowerment and socioeconomic security) across six Asia-Pacific countries: Australia, Hong Kong, Japan, South Korea, Taiwan, and Thailand.

**Methods:**

Population surveys were undertaken using a validated instrument in 2009-10, with sample sizes around 1000 in each country. The four SDH were analysed using multivariate binomial logistic regression to identify socio-demographic predictors in each country.

**Results:**

Low socio-economic security was associated with low income in all six study countries and with poor subjective health in Japan, South Korea and Thailand and with being married or cohabiting in Australia and Hong Kong. Low social cohesion was associated with low income in all countries and with undertaking household duties in South Korea, Thailand and Taiwan. Low social inclusion was associated with low income in Australia, South Korea and Taiwan and with poor subjective health in Australia, Japan and South Korea. Older people had lower social inclusion in Taiwan (50-59 years) and Hong Kong (retired), younger people in Japan and South Korea (20-29 years in both countries) and younger and middle-aged people in Australia. Low social empowerment was associated with low income in Australia, Thailand and Taiwan, with being aged 60 years or over in Australia, Hong Kong and South Korea, and over 50 years in Thailand.

**Conclusions:**

This paper provides baseline measures for identifying where and how policy should be altered to improve the SDH. Furthermore, these data can be used for future policy evaluation to identify whether changes in policy have indeed improved the SDH, particularly for marginalised and vulnerable populations.

## Introduction

In 2011, we published findings on the social determinants of health (SDH) in Australia, using social quality as our conceptual framework [1]. We assessed the levels and socio-demographic predictors of four concepts related directly to the SDH: socio-economic security, social inclusion, social cohesion and social empowerment. Taken together, we argued that these four concepts, emanating from social quality theory, represent a multidimensional and comprehensive toolkit for assessing the SDH. The current paper extends our previous analysis by providing comparative findings for these four concepts across six Asia-Pacific countries, namely Australia, Hong Kong, Japan, South Korea, Taiwan and Thailand. Data were collected in all six countries using the same survey instrument (translated into each language) between 2009 and 2010. Although this is the first paper to present data on the SDH across these countries, we have published a comparative analysis on the equity of access the healthcare services within and across the countries [2]. 

In this paper, we aim to describe the similarities and differences in both levels and predictors of the SDH across the six countries. In so doing, we identify population groups with higher or lower levels of socio-economic security, social inclusion, social capital and/or social empowerment, and importantly, population groups with low levels of all of these SDH. Our comparative findings will allow regional and national public health agencies to identify policies which may work for particular population groups in individual countries or across the Asia-Pacific region, and then to monitor changes in the SDH over time using our survey instrument. 

## Background

The World Health Organisation (WHO) has urged governments around the world to focus public health policy, practice and research on the SDH in order to improve the health of the most vulnerable and marginalised groups [3–5]. The Commission on the Social Determinants of Health (CSDH) drew global attention to the multiple forms of oppression and disadvantage experienced by the most vulnerable members of society which lead to unacceptable inequities in health [3]. Indeed, Professor Michael Marmot recently referred to these health inequities as a “stain on our society” which require concerted political will and moral imperative to change [6]. In order for governments to reduce inequities in health within their countries, the CSDH called for a ‘joined up’, multi-sectoral approach which recognises the multidimensional nature of the problem. In this way, reducing health inequities requires additional action in spheres of government policy outside of healthcare, such as poverty reduction, welfare support, community development and other health promotion activities. The CSDH also builds on seminal multi-national agreements such as the Ottawa Charter [7], the Alma Ata Declaration [8] and the Bangkok Declaration [9] which also argue for ‘joined up government’ in order to improve the health of the most vulnerable groups in society. 

Recognition of the SDH, from a policy perspective, is essential for health sector policy decision-making [10] since health policies shape health systems, and consequently, the broader SDH. Despite the differences in political and economic climate in the countries under analysis, our findings highlight patterns of social quality which are amenable to policy responses. We argue that our data could be used as a means of deciding the most appropriate policy response for each country which includes, rather than excludes, socially marginalised population groups [11]. These findings should be of interest to those involved in health policy, but also in policy more generally because as we have identified, health is influenced by determinants outside of the health system [12]. 

Our previous paper argued that many conceptual frameworks currently used in public health research tend to be rather tunnel visioned, rather than focussing on multiple and diverse forms of disadvantage, and thus the complexity of the SDH [1]. For example, there are large amounts of research which provide evidence that certain population groups are more socially excluded [13], have lower levels of social capital [14], have poorer access to financial resources, health promoting or curative services [15] and that some groups are disempowered [16]. All of these factors have been shown to be SDH [3], in that higher levels of social inclusion, social capital, access to finance and services and empowerment are all ‘good for your health’; however, taken on their own, these studies are useful only in so far as they paint part of the picture as to both the problems and solutions for increasing the health of vulnerable groups. What they do not do is provide both a conceptual and methodological framework for linking these various concepts for the same population groups, which would then highlight the potentially multiple or cumulative ‘problems’ that certain population groups face, or the particular ‘problems’ that other groups face. This fragmented policy and research context has been described as ‘…a field of turbulent discourses; different problems, different analyses and different strategies sweeping across the policy field like storm clouds under time lapse photography (p. 22)” [17]. Research studies may highlight the need to implement policy to increase the social capital for particular groups, or to facilitate more socially inclusive policies or systems, but rarely can such studies (due to their conceptual limitations) provide evidence for policies and systems which attend to the multiplicity of needs highlighted by the CSDH. Therefore, research is required to unify theories and analyses that on their own, concentrate on one aspect of a dynamic and fragmented health and social policy context.

In our previous paper, we provided a detailed account of social quality theory, which we argued provides a comprehensive conceptual and methodological framework for measuring the SDH [1]. However, we provide a brief summary here in order to provide the necessary context for this paper. 

Social quality theory is gaining international recognition as an innovative theoretical and methodological tool for researchers and policy makers in social policy and political science [18–27], although little attention has been given within public health research and policy. Social quality has been defined as “the extent to which people are able to participate in the social, economic life and development of their communities under conditions which enhance their wellbeing and individual potential (p. 3)” [27]. 

Social quality theory was initially developed by the European Network Indicators of Social Quality (ENISQ) [28]. The ENISQ developed indicators (or metrics) of social quality so that governments and researchers could assess social quality within and between societies or Nation States, using only routinely available data sources. Whilst this has benefits in terms of not needing to design and implement primary research, it also relies on existing datasets, which are often collected for administrative purposes and are often relatively old. Therefore, we used the indicators to develop a new social quality questionnaire to measure social quality, which was translated into relevant languages in order to allow data collection in the study countries. In this way, we have advanced the methodological and practical aspects of social quality theory by providing researchers and policy makers with a readily available instrument to measure social quality in their jurisdictions. 

Social quality theory has both ideological and methodological underpinnings. In terms of its underlying ideology, social quality theory argues that there are four key *normative factors* that determine the quality of the social structures, policies and relationships within a society: social justice; solidarity; equal value of all humans; and human dignity [29]. A society can be judged according to these normative factors, both in a global sense (i.e. how good is the social quality of a particular society) but also in terms of the specific normative factors (i.e. which factors require policy response in a particular society). However, on their own, these normative factors are not easily operationalised and do not have a methodological framework. Therefore, within social quality theory, there are a set of *conditional factors* which are aimed at rendering the normative factors ‘researchable’. The four conditional factors are socio-economic security (linked to social justice), social cohesion (linked to solidarity), social inclusion (linked to equal value) and social empowerment (linked to human dignity). These four conditional factors were measured in each of the six study countries using the newly developed social quality survey. 

Socio-economic security is concerned with the extent to which people or groups have access to, utilisation of and successful outcomes related to a variety of resources over time. These resources may be related to, among other things, finance, housing, healthcare, employment and education. This domain has great historical credence in public health policy and practice in terms of the importance of such factors in shaping inequalities in health and inequities in health care. Huge effort has been put into both public health policy [3,30,31] and research around understanding the causes and mechanisms of socio-economic inequalities in health, with most authors regarding it as a key SDH [32–35].

Social cohesion relates to the extent to which people and groups share social relations. Such relations may refer to shared identities, values and norms. This domain relates closely to issues of solidarity and trust, which are again, particularly important in terms of public health [16,36,37]. In many ways, this domain relates to the concept of social capital, which is now commonplace in public health policy and research [38–40], although has its roots in sociological theory [41–45]. Indeed, early sociologists such as Durkheim argued for the centrality of social cohesion for protecting health [46] and contemporary sociologists such as Giddens and Luhmann argue that trust and social networks are the glue that hold society together, providing existential security, thereby protecting mental health [47–50]. 

Social inclusion, is in many ways, similar to social cohesion, although the difference is that social inclusion is related to the extent to which people and groups have access to and are integrated into the different institutions and social relations of ‘everyday life’. This domain relates to the extent to which people and groups ‘feel part of’ or included in society, at an everyday level, and thus attempts to integrate dualistic processes at the level of systems (i.e. institutions and social systems) and individuals. In so doing, it extends Parsons’ notions of social systems by seeing their interconnectedness with individual lifeworlds [51], which Giddens called the duality of structure [52]. In this way, the domain of social inclusion fits neatly with system/lifeworld theories expounded by Habermas [53] and structure/agency argued by Giddens [49,52,54] and Archer [55] since it encompasses both individual and system-wide action. In addition, empirically-based public health research also provides empirical evidence on the links between social inclusion and health [56–58], showing its centrality as a SDH.

Social empowerment relates to the extent to which the personal capabilities of individual people are enhanced by social relations, culminating in individuals feeling empowered within their country. In many ways, this builds on both social cohesion and social inclusion, revealing the integrated nature of social quality theory. In this way, this domain takes concepts of social inclusion and cohesion, and explores the enabling factors which empower people to act as social agents. This domain builds on, and empirically develops, notions of reflexivity outlined by Beck [59–61] and Giddens [50] and extends the current evidence base on the positive effects of empowerment on both individual and public health [62–64].

As can be seen in this brief overview, the multi-dimensional and multi-level approach represents an advancement of public health policy and research, which is not solely aimed at either individuals or systems, but instead realises the intimate linkages between structure and agency and thus aims at understanding both within the same theoretical framework. The four conditional factors within social quality have all been shown individually to lead to better health [1,3], and as such are regarded as SDH, although have not been brought together into a single theoretical framework.The long-term aim of developing and implementing social quality theory is to enhance the social quality of peoples’ lives (especially vulnerable groups), but as already stated, we first need to have empirical data on the domains of social quality (and the groups who have lower social quality) before we can inform changes in policy and/or practice. The aim of this paper is to describe the patterns within each country with regards to social quality (as measures of the SDH) and to identify both consistent and divergent patterns between countries. This paper therefore represents baseline data from which the effectiveness of any future policy initiatives in these countries can be assessed.

## Methods

The data presented in this manuscript come from a larger survey designed to investigate social quality across six Asia-Pacific counties: Australia, Hong Kong, Japan, South Korea, Taiwan, and Thailand. The survey was developed, and data collected, by academic representatives from each of the universities involved; Australia (Flinders University), Hong Kong (Chinese University Hong Kong), Japan (Chiba University), South Korea (Seoul National University), Taiwan (National Taiwan University), Thailand (King Prajadhipok Institute). 

The original indicators of social quality, developed by the ENISQ, were developed into a questionnaire by academics from all of the above Universities. All of the questions used in the questionnaire were either demographically relevant or related to any one of the four conditional factors of social quality (socio-economic security, social inclusion, social cohesion and social empowerment). Questions from pre-validated questionnaires, including the World Values Survey [65] and the General Social Survey [66], were also employed in the SQ survey since they had previously been validated. Initially, the survey consisted of 58 questions that were predominantly constituted by nominal and ordinal levels of measurement. Although the questionnaire up until this point had been comprehensively developed and validated, in particular, for face, content, and construct validity [67], we needed to verify that we had constructed a valid set of questions. 

The initial stages of validity checking involved collaborative efforts across the Asia-Pacific research team, which included numerous and extensive face-to-face discussions. Revision and modification of the questionnaire lasted for approximately three months, including meticulous discussions of the cultural relevance of each question. The final questionnaire was agreed upon between all international teams in July 2009, which was subsequently tested for both validity and reliability [25], including collaboration and agreement with the originators of the social quality indicators [68]. 

The final questionnaire had 50 questions, divided into the four conditional factors: 4 questions related to socio-economic security, 11 questions related to social inclusion, 5 questions related to social cohesion, 19 questions related to social empowerment, and 11 related to socio-demographics (the full questionnaire is available on-line – see Additional File 1). The survey was then translated into the language of the host country and validated within each country. Data were collected in each country between 2009 and 2010.

The authors undertook data collection in Australia only and therefore, this paper reports a secondary analysis of a combined dataset representing all countries involved. The merging and cleaning of the dataset was conducted by academics at Seoul National University. In addition, once we had a cleaned SPSS dataset, data were weighted for each country on the basis of age and sex, to mitigate potential bias in age-sex response rates. Details of method for selected countries represented in this paper are published elsewhere [1,69–72], although a summary of this is provided in Table 1. We also provide a methodological critique of the various surveys in the discussion section of the paper, inclusing issues realted to sampling methods and response rates. The merged dataset was then provided to all members of the Asia-Pacific research team for further analysis and publications. Further discussion of the methodological issues are provided in more detail as limitations in the conclusion of the paper.

**Table 1:** Comparison of survey methods for six countries.

	**Australia**	**Japan**	**South Korea**	**Hong Kong**	**Taiwan-Taipei**	**Thailand**
**Fieldwork Dates**	Sept. 2009	11 Sept. - 21 Sept. 2009	20 Oct. - 10 Nov., 2009	October 2009 -October 2010	6 Oct. - 16 Nov., 2009	20 Oct. - 10 Nov., 2009
**Population**	18 years old and over	19 years old and over	18 years old and over	18 years old or above	Taipei legal citizens who are 20 years old or older	18 years old and over
**Geographic coverage**	Nationwide	Nationwide	Nationwide	Hong Kong	Taipei	Nationwide
**Sampling method**	Stratified according to state population	Regionally stratified proportional random sampling	Multi – Stages Sampling	Quota sampling based on the three age ranges	Central Location Quota Sampling	Multi – Stages Sampling
**Fieldwork Methods**	Self-administered postal survey	Face to face interview	Face to face interview	Telephone interview	Face to face interview	Face to face interview
**Sample size**	1,044	1,006	1,200	681	1,200	1,200

Appropriate approvals were obtained within each country to undertake the individual surveys. The authors were granted ethics approval from Flinders University Social and Behavioural Research Ethics Committee to obtain and use the collected data for secondary analysis (project number 5221). 

### Data Analysis

For the regression models presented in this paper, four questions identified by the ENISQ in 2004 [68] as indicators of the four domains of social quality were used as dependent variables (i.e. one variable per domain of social quality). These are the same variables used in our previous paper on social quality in Australia [1]. In order to conduct binomial regression analysis, the original four dependent variables were recoded into new binary variables as outlined below.

#### Socio-economic security

Respondents’ financial situation in the previous 12 months was surveyed in all six countries of interest, by asking the question “*During the past year, did you*?”. Response categories provided were the same in all countries, including ‘Saved money’ (1), ‘Just got by’ (2), ‘Spent some savings’ (3), and ‘Spent savings and borrowed money’ (4). Responses were dichotomised to represent householders who saved money or managed to get by (1,2) versus householders who spent some savings or spent savings and borrowed money (3,4).

#### Social cohesion

The outcome variable for social cohesion was generated from multiple survey items enquiring about respondents’ membership in several institutions, groups and/or organisations, with a statement “For each of the following organisations, please indicate your membership status”. In order to maintain cultural relevance, the specific types of organisations differed between countries. For Australia, the separate items subsumed under membership were questions relating to membership of four organisations, groups and institutions, including ‘church or religious organization’, ‘sport or recreational organization’, ‘cultural organization’ and ‘community-based organization’. The response categories provided for these items were either ‘member’ or ‘don’t belong’. To generate the outcome variable ‘membership’, respondents who reported being a member in at least one of the 4 possible organizations were classed as ‘member’, versus participants who did not report membership in any of the 4 organizations. This resulted in a binary membership outcome variable for Australia with two levels, member versus not member. In Hong Kong, 10 survey items were combined to form a membership status outcome variable, each of which survey participants responded to based on three possible response options, which were ‘active member’, ‘inactive member’ or ‘don’t belong’. The 10 survey items subsumed to generate membership status addressed respondents’ membership in ‘church or religious organization’, ‘sport or recreational organization’, ‘cultural organization’, ‘labor union’, ‘political party’, ‘occupational organization’, ‘NGO’, ‘School-related organization’, ‘Familial organization’ and ‘other’. Active and inactive membership were combined and contrasted with ‘don’t belong’ responses, so that respondents who reported being an active or inactive member in at least one of the 10 possible organizations or groups were classified as ‘member’ and respondents who reported not belonging to any of the organizations listed received a label of not member. The same survey items, response options and coding processes were present for South Korea, Thailand and Taiwan, hence membership outcome variables in Hong Kong, South Korea, Taiwan and Thailand were identically formed and dichotomised. Respondents in Japan were asked to respond with ‘yes’ or ‘no’ when asked about membership in ‘church or religious organization’, ‘sport or recreational organization’, ‘political party’, ‘volunteer activity’, ‘neighbourhood community’ and ‘labor union or occupational association’. Membership was assigned to respondents who indicated membership in at least one of six organizations investigated as opposed to respondents who responded with ‘no’ to all of the organizations enquired about. 

#### Social Inclusion

Discrimination experience in the previous 12 months was generated from several separate items enquiring about different discrimination experiences, using the question “During the past 12 months, have you ever experienced discrimination against you due to any of the following reasons?”. The number of response items varied between countries, however survey respondents in all countries were provided with the same response options, namely ‘yes’ and ‘no’. Respondents therefore indicated whether or not they had experienced particular types of discrimination in the 12 months prior to survey completion, which resulted in a binary outcome variable for discrimination, which opposed respondents who had experienced at least one of the discrimination types listed in their particular country with respondents who had not experienced any of the discrimination types included in the survey in their country. In Australia, respondents’ discrimination variable was generated based on the 10 following types of discrimination: physical handicap, age, sexual harassment, gender, nationality, physical look, region of origin, criminal record, religion as well as an ‘other’ category. In Hong Kong, discrimination was generated based on 12 types of discrimination experience, which included all of the items listed in the Australian survey plus items enquiring about discrimination based on social status and educational degree. The same applied to Thailand and Taiwan. South Korean surveys included all of the items listed in Hong Kong, Thailand and Taiwan, except for the ‘other’ category. In Japan, discrimination experiences listed included all items enquired about in Hong Kong, apart from the item on sexual harassment and criminal record.

#### Social Empowerment

An outcome variable labelled ‘social empowerment’ was generated for each participant in all countries except Japan, where this question was not asked due to lack of cultural relevance (determined by our Japanese colleagues at Chiba University). The variable used in the regression models was the result of responses given to multiple separate survey items enquiring about different types of political participation or activity, by asking the question “Have you or would you participate in any of the political actions listed below?”. Across countries, the same items and response options were included in the survey, which facilitated identical coding processes to be carried out for all countries. In particular, five survey items were formulated to enquire about political participation, including ‘signing a petition’, ‘joining boycotts’, ‘joining demonstrations’, ‘joining strikes’ and ‘online political actions’. In all countries, survey participants were provided with one of three possible response options, namely ‘have done’, ‘might do’ and ‘never would’. To yield a binary outcome variable, response options ‘have done’ and ‘might do’ were combined and opposed with ‘never would’. As a result, respondents who responded with ‘have done/might do’ for at least one political participation item were grouped together and contrasted with respondents who stated that they would never participate in any of the activities listed.

Ten independent variables (sex, age, marital status, work status, income, financial situation in the last year, subjective health satisfaction, self-rated health, perception of importance of health, and chronic health condition) were tested against the four dependent variables based on previous studies linking their relevance to the social determinants of health [73–75]. In contrast to Australia and Japan, where annual household income was surveyed, financial information in Hong Kong and all other countries was based on monthly household income. Moreover, for Australia annual household income was divided into six sub-categories, whereas for all other countries income was divided into thirds (based on the frequency of respondents).

Data were analysed using the SPSS version 17.0 (SPSS Inc., Chicago, IL, USA). Binomial logistic regression models were used to investigate associations for all six countries [76]. Due to differences in data collection methods, and changes to survey questions to make them culturally relevant, a few of the independent variables were not available from some countries thus reducing the number of association tests performed. Goodness-of-fit for all models were checked [76,77]. The regression models presented in the paper give details of the statistically significant predictors of each of the four conditional factors of social quality within each country. We could not perform statistical tests allowing a comparison across the countries (mixed effects models) because of some of the slightly different predictor variables and sampling techniques. However, within the paper we provide tentative comparisons of the regression models across the countries in order to highlight possible similarities and differences requiring further research. Nevertheless, all comparisons made within the paper need to be interpreted with caution.

To ensure that there would be no redundant calculations during the multivariate analyses, collinearity diagnostics (using SPSS) were performed to check for variables that may have similar degrees of variance [78]. A tolerance value of ≤ 0.20 and a variance inflation factor of ≥10 were used to indicate a multi-collinearity problem [79]. Diagnostics were conducted for all regression models. 

## Results

This section of the paper provides statistical description and analysis of the data. One multivariate logistic regression model is presented for each country for each the four domains of social quality. Each of the regression models includes one social quality variable as the dependent variable (socio-economic security, social inclusion, social cohesion and social empowerment) and a number of socio-demographic and health-related variables as independent variables. Table 2 provides descriptive data on the main predictor variables within the regression models to highlight the similarities in respondents between the six countries. Provided are the total number of discrete responses for each of the socio-demographically oriented survey items in each country, as well as in brackets associated percentages based on the total sample size.

**Table 2:** Socio-demographic characteristics of survey samples in the 6 Asia-Pacific countries.

**Socio-demographic variable**	**Australia (%)**	**Hong Kong (%)**	**Japan (%)**	**South Korea (%)**	**Taiwan (%)**	**Thailand (%)**
***Sex***	***1039***	***681***	***1000***	***1006***	***1200***	***1200***
*Male*	462 (44.5)	313 (46.0)	482 (48.2)	494 (49.1)	569 (47.4)	581 (48.4)
*Female*	577 (55.5)	368 (54.0)	518 (51.8)	512 (50.9)	631 (53.6)	619 (51.6)
***Age group***	***868***	***674***	***1000***	***1006***	***1200***	***1200***
*< 20 years*	23 (2.6)	47 (7.0)	N/A	35 (3.4)	N/A	53 (4.4)
*20 - 29 years*	158 (18.3)	109 (16.1)	151 (15.1)	204 (20.3)	209 (17.4)	275 (22.9)
*30 - 39 years*	165 (19.0)	122 (18.1)	179 (17.9)	228 (22.7)	238 (19.8)	283 (23.6)
*40 - 49 years*	268 (19.3)	138 (20.5)	153 (15.3)	223 (22.2)	259 (21.6)	253 (21.1)
*50 - 59 years*	147 (16.9)	120 (17.8)	185 (18.5)	143 (14.2)	240 (20.0)	165 (13.7)
*60 years and above*	208 (24.0)	138 (20.5)	332 (33.2)	174 (17.3)	254 (21.2)	172 (14.2)
***Marital status***	***1040***	***680***	***1000***	***1006***	***1198***	***1196***
*Married/cohabitating*	653 (62.8)	405 (59.5)	735 (73.5)	666 (66.2)	677 (56.5)	747 (62.4)
*Separated/divorced/widowed*	184 (17.7)	54 (7.9)	118 (11.8)	79 (7.9)	130 (10.9)	144 (12.0)
*Never married*	203 (19.5)	222 (32.7)	147 (14.7)	261 (25.9)	390 (32.6)	306 (25.5)
***Work status***	***1032***	***681***	***1000***	***1006***	***1200***	***1196***
*Full time / Self-employed*	453 (43.9)	312 (45.8)	444 (44.4)	562 (55.9)	741 (61.7)	689 (57.6)
*Part time*	158 (15.3)	56 (8.3)	200 (20.0)	69 (6.9)	82 (6.9)	47 (3.9)
*Work without pay/student/unemployed*	134 (12.9)	116 (17.1)	115 (11.5)	131 (13.0)	95 (8.0)	358 (29.9)
*Retired / Pensioner*	228 (22.1)	112 (16.5)	62 (6.2)	37 (3.7)	161 (13.4)	23 (1.9)
*Household duties*	59 (5.7)	84 (12.4)	179 (17.9)	207 (20.5)	121 (10.1)	79 (6.6)

### Socio-economic security

#### Australia

Two socio-demographic predictors were found to be significant for spending savings [χ^2^(7)=26.18, p<.001, Nagelkerke R^2^=0.04], which were respondents’ marital status [Wald χ^2^(2)=11.15, p<.01] and annual household income [Wald χ^2^(5)=21.02, p<.01] (see Table 3). For income, higher annual household income was associated with a lower likelihood of having spent savings and/or borrowed money: relative to respondents in the lowest income category (less than $30,000 – all $ quoted in this paper are Australian Dollars), individuals with an income over $90,000 were much less likely to have spent savings and/or borrowed money (OR=0.35; 95% CI: 0.20-0.62, OR=0.50; 95% CI: 0.28-0.90, and OR=0.38; 95% CI: 0.21-0.68, respectively). In regards to marital status, separated, divorced or widowed individuals (OR=0.56; 95% CI: 0.36-0.86) as well as never married respondents (OR=0.59; 95% CI: 0.40-0.88) were less likely to have spent their savings and/or borrowed money than respondents who were married or cohabitating.

**Table 3:** Regression model for socio-economic security in Australia.

Predictor	OR (95% CI)	P
Marital status (Married, cohabitating)		
Divorced, separated, widowed	0.56 (0.36-0.86)	.008
Never married	0.59 (0.40-0.88)	.010
Annual household income (<$30,000)		
$30,000 - $59,999	0.81 (0.53-1.23)	.313
$60,000 - $89,999	0.75 (0.48-1.17)	.202
$90,000 - $119,999	0.35 (0.20-0.62)	<.001
$120,000 - $149,999	0.50 (0.28-0.90)	.020
$150,000 and above	0.38 (0.21-0.68)	.001

*Reference group in parentheses

#### Hong Kong

The same two variables with predictive qualities for spending savings in Australia were also found to be significant in Hong Kong [χ^2^(4)=58.88, p<.001, Nagelkerke R^2^=0.14], marital status [Wald χ^2^(2)=13.54, p<.01] and monthly household income [Wald χ^2^(2)=43.82, p<.001] (see Table 4). Similar to Australian respondents, higher household income was associated with a lower likelihood of having spent and/or borrowed money in Hong Kong. Relative to the third of respondents falling into the lowest income group, respondents in the middle third and upper third income groups were much less likely to have spent savings and/or borrowed money (OR=0.35; 95% CI: 0.22-0.55 and OR=0.18: 95% CI: 0.11-0.32, respectively). Not being married or cohabiting in Hong Kong was associated with spending in a way comparable to Australia: respondents who were separated, divorced or widowed were more than 60% less likely to have spent savings or spent and borrowed money than married or cohabiting individuals (OR=0.37; 95% CI: 0.17-0.79). Similar results were found for respondents who were never married (OR=0.49; 95% CI: 0.32-0.78).

**Table 4:** Regression model for socio-economic security in Hong Kong.

Predictor	OR (95% CI)	p
Marital status (Married, cohabitating)		
Divorced, separated, widowed	0.37 (0.17-0.79)	.010
Never married	0.49 (0.32-0.78)	.002
Monthly household income (Lower third)		
Middle third	0.35 (0.22-0.55)	<.001
Upper third	0.18 (0.11-0.32)	<.001

*Reference group in parentheses

#### Japan

As for Australia and Hong Kong, household income was significantly predictive of spending savings, although subjective health status was also predictive within the model in Japan [χ^2^(3)=73.30, p<.001, Nagelkerke R^2^=0.11] (see Table 5). As obtained for the previous countries, annual household income was associated with a lower likelihood of having spent savings and/or borrowed money [Wald χ^2^(2)=48.53, p<.001], with respondents in the middle third of income groups being more than 40%, and those in the upper third close to 80% less likely to have spent and/or borrowed money than individuals in the lowest annual household income group (OR=0.57; 95% CI: 0.41-0.78 and OR=0.22; 95% CI: 0.14-0.34, respectively). Perceiving one’s subjective health status not as good was associated with a higher likelihood of having spent and/or borrowed money [Wald χ^2^(1)=9.22, p<.01]: individuals who reported their health status as fair, bad or very bad were more than 60% more likely to have spent and/or borrowed money than individuals with a subjective health status of good or very good (OR=1.61; 95% CI: 1.18-2.19).

**Table 5:** Regression model for socio-economic security in Japan.

Predictor	OR (95% CI)	p
Annual household income (Lower third)		
Middle third	0.57 (0.41-0.78)	<.001
Upper third	0.22 (0.14-0.34)	<.001
Subjective health status ((Very) Good)		
Fair, bad, very bad	1.61 (1.18-2.19)	.002

*Reference group in parentheses

#### South Korea

The results obtained for South Korea mirrored the findings in Japan, with household income and subjective health status being significant predictors for spending savings [χ^2^(3)=50.13, p<.001, Nagelkerke R^2^=0.09] (see Table 6). Higher household income was associated with a smaller likelihood of having spent and/or borrowed money relative to respondents in the lower third [Wald χ^2^(2)=12.52, p<.01]. In particular, respondents in the upper third were around half as likely to have spent and/or borrowed money (OR=0.46; 95% CI: 0.29-0.71) than respondents in the lower third. The results for subjective health were comparable to those in Japan [Wald χ^2^(1)=27.16, p<.001]. South Korean respondents who reported their health as fair, bad or very bad were more than 160% more likely to have spent and/or borrowed money than respondents who perceived their health as good or very good (OR=2.62; 95% CI: 1.83-3.77).

**Table 6:** Regression model for socio-economic security in South Korea.

Predictor	OR (95% CI)	p
Monthly household income (Lower third)		
Middle third	0.85 (0.55-1.32)	.466
Upper third	0.46 (0.29-0.71)	<.001
Subjective health status ((Very) Good)		
Fair, bad, very bad	2.62 (1.83-3.77)	<.001

*Reference group in parentheses

#### Thailand

Three variables were found to be significant for spending savings [χ^2^(7)=62.05, p<.001, Nagelkerke R^2^=0.08], namely marital status [Wald χ^2^(2)=14.65, p<.01], work status [Wald χ^2^(4)=40.86, p<.001] and presence of a chronic condition [Wald χ^2^(1)=10.58, p<.01] (see Table 7). Separated, divorced and widowed respondents were more than 55% less likely (OR=0.44; 95% CI: 0.27-0.73), whereas never married respondents were approximately 40% less likely to have spent and/or borrowed money (OR=0.63; 95% CI: 0.44-0.88) than married or cohabitating individuals. Relative to respondents who reported to be working full time or self-employed, individuals who worked without pay, were unemployed or students were 2.5 times as likely to have spent and/or borrowed money (OR=2.56; 95% CI: 1.89-3.48). Having a chronic condition was associated with being more than twice as likely to have spent and/or borrowed money compared to those who reported the absence of a chronic condition (OR=2.34; 95% CI: 1.40-3.90). Being retired was marginally significant, with an odds ratio of 2.51 (95% CI: 0.98-6.41). 

**Table 7:** Regression model for socio-economic security in Thailand.

Predictor	OR (95% CI)	p
Marital status (Married, cohabitating)		
Divorced, separated, widowed	0.44 (0.27-0.73)	.002
Never married	0.63 (0.44-0.88)	.008
Work status (Full time/Self employed)		
Part time	1.56 (0.76-3.19)	.222
Work without pay, unemployed, student, other	2.56 (1.89-3.48)	<.001
Retired	2.51 (0.98-6.41)	.055
Household duties	0.85 (0.45-1.63)	.632
Chronic health problem (No)		
Yes	2.34 (1.40-3.90)	.001

*Reference group in parentheses

#### Taiwan

For Taiwan, spending savings was statistically significant [χ^2^(6)=23.27, p<.01, Nagelkerke R^2^=0.04] and two variables were (marginally) significant, those being respondents’ monthly household income [Wald χ^2^(2)=5.81, p<.06] and work status [Wald χ^2^(4)=15.42, p<.01] (see Table 8). Higher income was associated with a lower likelihood of having spent and/or borrowed money, as was being in full time employment or being self-employed. Specifically, respondents in the upper third of monthly household income were 40% less likely to have spent savings or spent savings and borrowed money than those in the lowest third (OR=0.60; 95% CI: 0.39-0.92). In regards to work status, individuals who worked without pay, were unemployed or students were more than three times as likely to have spent and/or borrowed money than those in full time employment or self-employed respondents (OR=3.08; 95% CI: 1.62-5.82).

**Table 8:** Regression model for socio-economic security in Taiwan.

Predictor	OR (95% CI)	p
Monthly household income (Lower third)		
Middle third	0.75 (0.48-1.17)	.203
Upper third	0.60 (0.39-0.92)	.018
Work status (Full time/Self employed)		
Part time	1.74 (0.91-3.32)	.091
Work without pay, unemployed, student, other	3.08 (1.62-5.82)	.001
Retired	1.58 (0.94-2.63)	.082
Household duties	0.91 (0.47-1.76)	.771

*Reference group in parentheses

### Social Cohesion

#### Australia

A significant social cohesion model was established for Australia [χ^2^(17)=128.60, p<.001, Nagelkerke R^2^=0.21] (see Table 9). Variables which contributed to the significance of the model were age [Wald χ^2^(5)=18.33, p<.01], work status [Wald χ^2^(4)=13.91, p<.01], annual household income [Wald χ^2^(5)=23.41, p<.001], respondents’ subjective health status [Wald χ^2^(1)=26.70, p<.001] and the presence of a chronic condition [Wald χ^2^(1)=21.98, p<.001]. Sex was marginally significant for predicting membership in at least one type of organisation or group [Wald χ^2^(1)=3.82, p<.06]. 

**Table 9:** Regression model for social cohesion in Australia.

Predictor	OR (95% CI)	P
Sex (Female)		
Male	0.71 (0.50-1.00)	.051
Age (60+)		
<20	0.09 (0.02-0.55)	.009
20-29	0.24 (0.11-0.52)	<.001
30-39	0.20 (0.09-0.44)	<.001
40-49	0.27 (0.12-0.59)	.001
50-59	0.30 (0.14-0.62)	.008
Work status (Full time/Self employed)		
Part time	1.27 (0.79-2.07)	.327
Working without pay, unemployed, student, other	3.00 (1.67-5.38)	<.001
Retired / Pensioner	1.23 (0.57-2.66)	.599
Household duties	0.98 (0.49-1.95)	.944
Annual household income (<$30,000)		
$30,000 - $59,999	1.39 (0.78-2.47)	.261
$60,000 - $89,999	1.11 (0.62-2.00)	.730
$90,000 - $119,999	1.74 (0.89-3.38)	.105
$120,000 - $149,999	4.82 (2.15-10.80)	<.001
$150,000 and above	2.67 (1.32-5.41)	.006
Subjective health status ((Very) Good)		
Fair, bad, very bad	0.35 (0.24-0.52)	<.001
Chronic health problem (No)		
Yes	2.71 (1.79-4.10)	<.001

*Reference group in parentheses

Males were approximately 30% less likely to indicate membership than females (OR=0.71; 95% CI: 0.50-1.00). The youngest group (< 20 years) were over 90% less likely (OR=0.09; 95% CI: 0.02-0.55), respondents between 20 and 29 years were around 75% less likely (OR=0.24; 95% CI: 0.11-0.52) and respondents between 30 and 39 years were almost 80% less likely (OR=0.20; 95% CI: 0.09-0.44) to report membership than the oldest group. Respondents who worked without pay, were unemployed or students were three times more likely to be members of organisation that those in full time employment or self-employment (OR=3.00; 95% CI: 1.67-5.38). The general pattern for household income was that a higher level of income was associated with a significantly higher likelihood for stating membership. For example, in relation to the lowest annual household income group (<$30,000), those with an income between $120,000 and $149,999 were close to five times more likely to report membership (OR=4.82; 95% CI: 2.15-10.80), although this association was reduced for the highest income group (OR=2.67; 95% CI: 1.32-5.41). Respondents who rated their subjective health as fair, bad or very bad were 65% less likely to report membership relative to respondents who rated their subjective health as good or very good (OR=0.35; 95% CI: 0.24-0.52). In contrast, having a chronic condition was associated with an almost three-fold increase in the likelihood of stating membership as opposed to not having a chronic condition (OR=2.71; 95% CI: 1.79-4.10).

#### Hong Kong

For Hong Kong, a significant model for social cohesion was established [χ^2^(4)=77.00, p<.001, Nagelkerke R^2^=0.16], which was based on marital status [Wald χ^2^(2)=52.33, p<.001] and household income [Wald χ^2^(2)=20.46, p<.001] (see Table 10). Compared to the lowest income group, respondents in the middle cohort were almost twice as likely to indicate membership (OR=1.98; 95% CI: 1.32-2.98), while respondents in the highest income group were more than 2.5 times as likely (OR=2.59; 95% CI: 1.69-3.98). In regards to marital status, separated, divorced or widowed individuals were almost 13.5 times more likely to report being a member in at least one type of group or organisation than respondents who indicated to be married or cohabiting (OR=13.43; 95% CI: 5.57-32.38), while the group comprising never married individuals was more than 2.5 times more likely to report membership than married or cohabiting respondents (OR=2.57; 95% CI: 1.80-3.57).

**Table 10:** Regression model for social cohesion in Hong Kong.

Predictor	OR (95% CI)	P
Marital status (Married, Cohabitated)		
Separated, widowed, divorced	13.43 (5.57-32.38)	<.001
Never married	2.57 (1.80-3.67)	<.001
Monthly household income (Lower third)		
Middle third	1.98 (1.32-2.98)	.001
Upper third	2.59 (1.69-3.98)	<.001

*Reference group in parentheses

#### Japan

A significant model for social cohesion was not established for Japan.

#### South Korea

Regression analysis yielded a two-factor model for social cohesion in South Korea [χ^2^(6)=42.41, p<.001, Nagelkerke R^2^=0.07], including respondents’ work status [Wald χ^2^(4)=17.49, p<.01] and household income [Wald χ^2^(2)=22.41, p<.001] (see Table 11). Relative to respondents in full time employment or who were self-employed, respondents who were employed part time were almost 60% less likely to report being a member in at least one group or organisation (OR=0.37; 95% CI: 0.22-0.64). Respondents reporting household duties were more than 30% less likely to indicate membership (OR=0.69; 95% CI: 0.47-1.02). Higher income was associated with a markedly higher likelihood of reporting membership, as evidenced by respondents in the upper income third being 2.5 times as likely (OR=2.5; 95% CI: 1.71-3.66) as the reference group (lower third) to report membership in any organisation or institution. 

**Table 11:** Regression model for social cohesion in South Korea.

Predictor	OR (95% CI)	p
Work status (Full time/Self employed)		
Part time	0.37 (0.22-0.64)	<.001
Work without pay, unemployed, student, other	0.69 (0.42-1.12)	.129
Retired	1.89 (0.69-5.15)	.215
Household duties	0.69 (0.47-1.02)	.060
Monthly household income (Lower third)		
Middle third	1.31 (0.89-1.93)	.178
Upper third	2.50 (1.71-3.66)	<.001

*Reference group in parentheses

#### Thailand

Mirroring the model obtained for South Korea, membership in Thailand was predicted by two factors [χ^2^(6)=21.20, p<.01, Nagelkerke R^2^=0.03], also based on respondents’ work status [Wald χ^2^(4)=12.19, p<.05] and the marginally significant predictor of household income [Wald χ^2^(2)=5.63, p=.06] (see Table 12). Compared to respondents in full time employment or who were self-employed, retired individuals were over 3 times more likely to indicate membership (OR=3.34; 95% CI: 1.37-8.11), whereas respondents reporting household duties were almost half as likely to report membership (OR=0.56; 95% CI: 0.31-0.99). Respondents in the upper third of household income were almost 50% more likely to indicate membership in at least one organisation, group or institution compared to respondents whose household income fell into the lower third (OR= 1.41; 95% CI: 1.04-1.93). 

**Table 12:** Regression model for social cohesion in Thailand.

Predictor	OR (95% CI)	P
Work status (Full time / Self employed)		
Part time	1.27 (0.68-2.36)	.451
Work without pay, unemployed, student, other	0.96 (0.72-1.28)	.770
Retired	3.34 (1.37-8.11)	.008
Household duties	0.56 (0.31-0.99)	.048
Monthly household income (Lower third)		
Middle third	1.07 (0.78-1.47)	.682
Upper third	1.41 (1.04-1.93)	.028

*Reference group in parentheses

#### Taiwan

Membership in Taiwan was predicted by three variables [χ^2^(7)=28.25, p<.001, Nagelkerke R^2^=0.05], sex [Wald χ^2^(1)=6.04, p<.05], marginally by work status [Wald χ^2^(4)=9.24, p<.06] and household income [Wald χ^2^(2)=11.49, p<.01] (see Table 13). Male respondents were approximately 40% less likely than female respondents to report membership (OR=0.61; 95% CI: 0.41-0.90). For work status, two category levels emerged as marginally significant, and one as significant. Respondents who were employed part-time as well as those who work without pay, unemployed or students were around half as likely to report membership than full time employed or self-employed individuals (OR=0.52; 95% CI: 0.27-0.99 and OR=0.52; 95% CI: 0.26-1.02, respectively). A similar odds ratio was found for respondents reporting household duties (OR=0.51; 95% CI: 0.27-0.97). The general pattern for income resembled findings for other countries in which income emerged as a significant predictor, where higher income was associated with an increased likelihood for indicating membership in at least one type of group or organisation. Respondents in the upper income third were more than twice as likely to report membership than respondents in the lowest income group (OR=2.17; 95% CI: 1.34-3.50).

**Table 13:** Regression model for social cohesion in Taiwan.

Predictor	OR (95% CI)	P
Sex (Female)		
Male	0.61 (0.41-0.90)	.014
Work status (Full time/Self employed)		
Part time	0.52 (0.27-0.99)	.050
Work without pay, unemployed, student, other	0.52 (0.26-1.02)	.056
Retired	0.94 (0.52-1.70)	.830
Household duties	0.51 (0.27-0.97)	.039
Monthly household income (Lower third)		
Middle third	1.00 (0.65-1.55)	.992
Upper third	2.17 (1.34-3.50)	.002

*Reference group in parentheses

### Social inclusion

#### Australia

A significant model for social inclusion was established [χ^2^(13)=112.13, p<.001, Nagelkerke R^2^=0.19]. The model included five significant predictor variables, namely sex [Wald χ^2^(1)=3.83, p=.05], age [Wald χ^2^(5)=38.61, p<.001], annual household income [Wald χ^2^(5)=27.08, p<.001], respondents’ subjective health status [Wald χ^2^(1)=6.74, p<.01] and the presence of a chronic condition [Wald χ^2^(1)=27.07, p<.001] (see Table 14). Male respondents were approximately 30% less likely to have experienced at least one type of discrimination compared to females (OR=0.71; 95% CI: 0.50-1.00). As age increased, the association with exposure to discrimination decreased. Relative to those aged 60 years or older, individuals <20 years and between 20 and 29 years were approximately 11 times (OR=10.71; 95% CI: 2.02-56.87) and 5 times (OR=5.15; 95% CI: 2.83-9.37) more likely to report discrimination, respectively. Respondents earning between $60,000 and $89,999 were more than 50% less likely to have experienced at least one type of discrimination than those earning less than $30,000 (OR=0.43; 95% CI: 0.23-0.81). In regards to health status, individuals who perceived their own health as being fair, bad or very bad were almost 70% more likely to have experienced at least one type of discrimination than individuals who rated their own health as good or very good (OR=1.67; 95% CI: 1.13-2.45). Respondents who reported a chronic condition were almost three times as likely as those without (OR=2.82; 95% CI: 1.91-4.17) to have experienced discrimination.

**Table 14:** Regression model for social inclusion in Australia.

Predictor	OR (95% CI)	p
Sex (Female)		
Male	0.71 (0.5-1.00)	.050
Age (60+)		
<20	10.71 (2.02-56.87)	.005
20-29	5.15 (2.83-9.37)	<.001
30-39	2.38 (1.32-4.29)	.004
40-49	2.06 (1.15-3.71)	.015
50-59	1.21 (0.65-2.26)	.540
Annual household income (<$30,000)		
$30,000 - $59,999	1.58 (0.92-2.73)	.098
$60,000 - $89,999	0.43 (0.23-0.81)	.009
$90,000 - $119,999	0.69 (0.35-1.34)	.268
$120,000 - $149,999	0.71 (0.34-1.48)	.366
$150,000 and above	0.81 (0.40-1.65)	.564
Subjective health status ((Very) Good)		
Fair, bad, very bad	1.67 (1.13-2.45)	.009
Chronic health problem (No)		
Yes	2.82 (1.91-4.17)	<.001

*Reference group in parentheses

#### Hong Kong

For social inclusion in Hong Kong, a one-factor model was found [χ^2^(4)=17.32, p<.01, Nagelkerke R^2^=0.04], with only respondents’ work status emerging as a significant indicator of having experienced at least one type of discrimination in the previous 12 months [Wald χ^2^(4)=13.89, p<.01] (see Table 15). Compared to individuals who were in full time employment or self-employed, retired respondents were 70% less likely to have reported at least one type of discrimination experience (OR=0.30; 95% CI: 0.14-0.61). 

**Table 15:** Regression model for social inclusion in Hong Kong.

Predictor	OR (95% CI)	p
Work status (Full time/Self-employed)		
Part time	1.38 (0.73-2.58)	.319
Work without pay, unemployed, student, other	0.96 (0.58-1.60)	.879
Retired / Pensioner	0.30 (0.14-0.61)	.001
Household duties	0.74 (0.40-1.36)	.332

*Reference group in parentheses

#### Japan

Similar to some of the findings from Australia and Hong Kong, a significant social inclusion model was established for Japan [χ^2^(9)=33.09, p<.001, Nagelkerke R^2^=0.06] based on three predictor variables: age [Wald χ^2^(4)=13.73, p<.01], work status [Wald χ^2^(4)=13.92, p<.01] and subjective health status [Wald χ^2^(1)=7.31, p<.01] (see Table 16). Taking the oldest cohort (60 years or older) as reference category, individuals aged 20-29 years and those aged 30-39 years were more than twice as likely to have experienced at least one type of discrimination (OR=2.29; 95% CI: 1.32-3.98 and OR=2.04; 95% CI: 1.16-3.60, respectively). Individuals in part-time employment were almost twice as likely to have experienced discrimination than full-time employed or self-employed respondents (OR=1.93; 95% CI: 1.24-2.99). Respondents whose own health status as fair, bad or very bad was associated with a higher likelihood of reporting a discrimination experience compared to individuals whose subjective health was rated good or very good (OR=1.71; 95% CI: 1.16-2.52).

**Table 16:** Regression model for social inclusion in Japan.

Predictor	OR (95% CI)	p
Age (60+ years)		
20-29	2.29 (1.32-3.98)	.003
30-39	2.04 (1.16-3.60)	.014
40-49	1.19 (0.63-2.24)	.599
50-59	1.11 (0.61-2.04)	.736
Work status (Full time/Self employed)		
Part time, contract, freelance	1.93 (1.24-2.99)	.004
Work without pay, unemployed, student, other	1.32 (0.72-2.43)	.372
Retired	0.71 (0.25-2.01)	.514
Household duties	0.75 (0.42-1.32)	.313
Subjective health status ((Very) Good)		
Fair, bad, very bad	1.71 (1.16-2.52)	.007

*Reference group in parentheses

#### South Korea

A four-factor model was found for social inclusion in South Korea [χ^2^(9)=63.92, p<.001, Nagelkerke R^2^=0.11] (see Table 17). The model was based on age [Wald χ^2^(5)=20.55, p<.01], household income [Wald χ^2^(2)=8.49, p<.05], subjective health status [Wald χ^2^(1)=21.72, p<.001] as well as presence of a chronic condition [Wald χ^2^(1)=6.77, p<.01]. Compared to the oldest cohort (60+ years), respondents aged 20-29 years were almost three times more likely to have reported at least one type of discrimination experience (OR=2.83; 95% CI: 1.56-5.14). Respondents in the middle income group were approximately 50% less likely (OR=0.51; 95% CI: 0.31-0.85) and respondents in the highest income group were approximately 40% less likely (OR=0.63; 95% CI: 0.41-0.96) to have experienced at least one type of discrimination than those in the lowest income group. Respondents with fair, bad or very bad perceived health were approximately 2.5 times more likely to have had discrimination experience in the previous 12 months compared to respondents with good or very good perceived health (OR=2.49; 95% CI: 1.70-3.65) and not having a chronic condition (OR=2.47; 95% CI: 1.25-4.89). 

**Table 17:** Regression model for social inclusion in South Korea.

Predictor	OR (95% CI)	P
Age (60+)		
<20	1.14 (0.33-3.87)	.840
20-29	2.83 (1.56-5.14)	.001
30-39	1.43 (0.79-2.57)	.236
40-49	0.86 (0.46-1.60)	.631
50-59	1.42 (0.76-2.67)	.269
Monthly household income (Lower third)		
Middle third	0.51 (0.31-0.85)	.009
Upper third	0.63 (0.41-0.96)	.031
Subjective health status ((Very) Good)		
Fair, bad, very bad	2.49 (1.70-3.65)	<.001
Chronic health problem (No)		
Yes	2.47 (1.25-4.89)	.009

*Reference group in parentheses

#### Thailand

No significant model for social inclusion was obtained for Thailand. 

#### Taiwan

A significant model for social inclusion was found for Taiwan [χ^2^(6)=26.36, p<.001, Nagelkerke R^2^=0.04]. Two variables emerged as significant indicators, which were age [Wald χ^2^(4)=9.97, p<.05] and household income [Wald χ^2^(2)=13.86, p<.01] (see Table 18). Compared to the oldest cohort (60+ years), individuals between 50 and 59 years of age were approximately 70% more likely to have reported at least one type of discrimination experience (OR=1.69; 95% CI: 1.05-2.72). Individuals with a household income in the middle third were more than 50% less likely to have had at least one type of discrimination experience than individuals in the lowest income group (OR=0.47; 95% CI: 0.30-0.73).

**Table 18:** Regression model for social inclusion in Taiwan.

Predictor	OR (95% CI)	P
Age (60+)		
20-29	1.18 (0.68-2.05)	.554
30-39	0.79 (0.46-1.34)	.374
40-49	1.21 (0.74-1.97)	.444
50-59	1.69 (1.05-2.72)	.032
Monthly household income (Lower third)		
Middle third	0.47 (0.30-0.73)	.001
Upper third	1.06 (0.75-1.51)	.734

*Reference group in parentheses

### Social empowerment

#### Australia

A significant model for social empowerment in Australia was found, containing five indicators [χ^2^(15)=96.20, p<.001, Nagelkerke R^2^=0.29]. Variables which contributed to the significance of the model were age [Wald χ^2^(4)=15.76, p<.01], work status [Wald χ^2^(4)=18.43, p<.01], annual household income [Wald χ^2^(5)=21.69, p<.01], respondents’ subjective health status [Wald χ^2^(1)=11.44, p<.01] and the presence of a chronic condition [Wald χ^2^(1)=5.42, p<.05] (see Table 19). Respondents aged 30-39 years were approximately 4.4 times more likely to have participated in political actions (OR=4.37; 95% CI: 1.12-15.75), and respondents aged 40-49 years were approximately 17.5 times as likely as the oldest cohort (OR=17.46; 95% CI: 2.53-120.66). Compared to full-time employed or self-employed respondents,those reporting household duties were approximately 80% less likely to report political activity (OR=0.19; 95% CI: 0.07-0.53). In comparison to those in the lowest income group (<$30,000), those between $60,000-$89,999 were approximately13 times more likely (OR=12.93; 95% CI: 3.32-50.36) and those between $120,000-$149,999 were approximately 22 times more likely (OR=22.42; 95% CI: 2.25-223.20) to participate in political activities. Respondents who perceived their health as being fair, bad or very bad were almost 70% less likely to have participated in political activities than people with subjective health as good or very good (OR=0.33; 95% CI: 0.17-0.62). Having a chronic condition was associated with being approximately 2.5 times more likely to have participated in political activities (OR=2.47; 95% CI: 1.15-5.27).

**Table 19:** Regression model for social empowerment in Australia.

Predictor	OR (95% CI)	p
Age (60+)		
20-29	1.10 (0.36-3.35)	.864
30-39	4.37 (1.21-15.75)	.024
40-49	17.46 (2.53-120.66)	.004
50-59	2.55 (0.78-8.35)	.122
Work status (Full time/Self employed)		
Part time	2.09 (0.69-6.31)	.190
Working without pay, unemployed, student, other	3.61 (0.79-16.42)	.097
Retired / Pensioner	3.18 (0.97-10.50)	.057
Household duties	0.19 (0.07-0.53)	.002
Annual household income (<$30,000)		
$30,000 - $59,999	1.46 (0.62-3.43)	.389
$60,000 - $89,999	12.93 (3.32-50.36)	<.001
$90,000 - $119,999	2.88 (0.81-10.24)	.101
$120,000 - $149,999	22.42 (2.25-223.20)	.008
$150,000 and above	4.13 (0.91-18.82)	.067
Subjective health status ((Very) Good)		
Fair, bad, very bad	0.33 (0.17-0.62)	.001
Chronic health problem (No)		
Yes	2.47 (1.15-5.27)	.020

*Reference group in parentheses

#### Hong Kong

The analysis for Hong Kong returned a significant model with two significant predictors [χ^2^(7)=49.89, p<.001, Nagelkerke R^2^=0.10]; age [Wald χ^2^(5)=23.30, p<.001] and marital status [Wald χ^2^(2)=15.12, p<.01] (see Table 20). Relative to respondents in the oldest age group (60+ years), statistically significant odds ratios included 2.26 (95% CI: 1.35-3.80) for 50-59 year olds, 2.49 (95% CI: 1.46-4.26) for 30-39 year olds and 2.69 for 40-49 year olds (95% CI: 1.63-4.45). In regards to marital status, being married or cohabiting as well as being separated, divorced or widowed was associated with being around 70% less likely to have participated in political activities as compared with those who were never married (OR=0.30; 95% CI: 0.16-0.56 and OR=0.25; 95% CI: 0.11-0.58, respectively).

**Table 20:** Regression model for social empowerment in Hong Kong.

Predictor	OR (95% CI)	p
Age (60+)		
<20	0.98 (0.40-2.44)	.972
20-29	1.04 (0.47-2.27)	.928
30-39	2.49 (1.46-4.26)	.001
40-49	2.69 (1.63-4.45)	<.001
50-59	2.26 (1.35-3.80)	.002
Marital status (Never married)		
Married, cohabitating	0.30 (0.16-0.56)	<.001
Separated, widowed, divorced	0.25 (0.11-0.58)	.001

*Reference group in parentheses

#### South Korea

A significant model for social empowerment in South Korea included two variables [χ^2^(6)=40.01, p<.001, Nagelkerke R^2^=0.05], namely age [Wald χ^2^(5)=34.57, p<.001] and sex (marginally significant) [Wald χ^2^(1)=3.74, p<.06] (see Table 21). Males were close to 30% more likely than females to indicate having participated in political actions (OR=1.29; 95% CI: 1.00-1.66). Relative to the oldest age cohort (60+ years), respondents aged between 20 and 49 years were around 2.5 (95% CI: 1.59-3.65 for 20-29 years, 95% CI: 1.68-3.79 for 30-39 years, and 95% CI: 1.68-3.81 for 40-49 years) times more likely to state that they have participated in political activities. 

**Table 21:** Regression model for social empowerment in South Korea.

Predictor	OR (95% CI)	p
Sex (Female)		
Male	1.29 (1.00-1.66)	.053
Age (60+)		
<20	1.21 (0.58-2.51)	.616
20-29	2.41 (1.59-3.65)	<.001
30-39	2.53 (1.68-3.79)	<.001
40-49	2.53 (1.68-3.81)	<.001
50-59	1.33 (0.85-2.07)	.215

*Reference group in parentheses

#### Thailand

In Thailand, a significant model for social empowerment was established [χ^2^(8)=36.49, p<.001, Nagelkerke R^2^=0.05] based on age [Wald χ^2^(5)=14.86, p<.05], household income [Wald χ^2^(2)=15.92, p<.001] and subjective health [Wald χ^2^(1)=4.23, p<.05] (see Table 22). Relative to the oldest age cohort (60+ years), younger groups were around twice as likely to have participated in political actions. Specifically, odds ratios ranged from 1.96 (95% CI: 1.07-3.56) for 20-29 year olds, 2.04 (95% CI: 1.14-3.68) for 30-39 year olds, and 2.36 (95% CI: 1.32-4.21) 40-49 year olds. Taking the lowest income group as reference category, higher income was associated with an increased likelihood for political participation, as evidence through respondents in the upper third being more than twice as likely to have participated in political actions (OR=2.10; 95% CI: 1.44-3.06). Counter to findings in Australia, rating one’s subjective health as fair, bad or very bad was associated with a 40% higher likelihood for political participation than when health is perceived as good or very good (OR=1.42; 95% CI: 1.02-1.97).

**Table 22:** Regression model for social empowerment in Thailand.

Predictor	OR (95% CI)	p
Age (60+)		
<20	2.03 (0.83-4.94)	.119
20-29	1.96 (1.07-3.56)	.028
30-39	2.04 (1.14-3.68)	.017
40-49	2.36 (1.32-4.21)	.004
50-59	0.97 (0.48-1.98)	.942
Monthly household income (Lower third)		
Middle third	1.30 (0.87-1.97)	.205
Lower third	2.10 (1.44-3.06)	<.001
Subjective health condition ((Very) Good)		
Fair, bad, very bad	1.42 (1.02-1.97)	.040

*Reference group in parentheses

#### Taiwan

A significant model for social empowerments was established for Taiwan [χ^2^(4)=23.68, p<.001, Nagelkerke R^2^=0.04, including household income [Wald χ^2^(2)=16.14, p<.001] and marital status [Wald χ^2^(2)=6.93, p<.05] (see Table 23). Not being married or cohabiting was associated with being less likely to report political participation, as evidenced by a 25-40% lower likelihood obtained for individuals who reported being separated, divorced or widowed (OR=0.61; 95% CI: 0.38-0.99) as well as those who stated to have never been married (OR=0.72; 95% CI: 0.53-0.98) relative to those married or cohabitating. Relative to respondents in the lowest income group, individuals in the middle third were close to 40% less likely (OR=0.64; 95% CI: 0.45-0.92) to report they have participated in political actions. In contrast, a marginally significant result was obtained for respondents whose household income fell into the upper third, who were close to 40% more likely to report political participation (OR=1.36; 95% CI: 0.99-1.86). 

**Table 23:** Regression model for social empowerment in Taiwan.

Predictor	OR (95% CI)	p
Marital status (Married, cohabitating)		
Separated, widowed, divorced	0.61 (0.38-0.99)	.045
Never married	0.72 (0.53-0.98)	.035
Monthly household income (Lower third)		
Middle third	0.64 (0.45-0.92)	.016
Upper third	1.36 (0.99-1.86)	.055

*Reference group in parentheses

## Discussion

The main aim of this paper was to provide an analysis of the SDH in six Asia-Pacific countries in order to compare and contrast the predictors and vulnerable populations. We fulfilled this aim by using comparative data from population surveys undertaken in each country which used social quality as their conceptual framework. In particular, we undertook logistic regression analyses of the four conditional factors within social quality: socio-economic security, social inclusion, social cohesion and social empowerment. Before summarising and discussing our overall findings, we provide a methodological critique of our study in order for readers to interpret our findings appropriately.

### Methodological critique

The first potential limitation relates to the different survey administration methods across the countries (see Table 1). The survey was administered face-to-face in Japan, South Korea, Taiwan and Thailand, by telephone in Hong Kong and mail in Australia. Face-to-face administration would be the ‘gold standard’ because of higher response rates although this method may potentially incur interviewer bias [67]. The sheer geographical size of Australia prohibited face-to-face survey administration and other than random number dialling, we do not have access to an up-to-date database of telephone numbers to allow a national telephone survey. In Hong Kong, the telephone administration was deemed the most practical solution. Although all methods of survey administration have their strengths and weaknesses, we believe that the different administration methods in Hong Kong and Australia still provide adequately comparative data, since the actual questionnaires administered were the same (but in appropriate languages).

The second potential limitation relates to response rates for the surveys. The surveys were administered by nationally and internationally reputable market research companies in most countries, who do not provide details on response rates. In South Korea and Taiwan the survey was administered by Gallup, and in Japan, it was administered by Nippon Research Centre Ltd. In Thailand it was administered by the Thai National Statistical Office who regularly undertake national surveys of this nature. In Hong Kong, the quota sampling involved potential participants identifying themselves to researchers on the basis of posters in the community and then the researchers made sure the final sample was representative on the basis of age and sex. All of these surveys involved non-random quota sampling which provides a representative sample, but no details of response rates which is a recognised potential limitation of the data in this paper. In Australia, response rate was 24% (1044/4362). We have argued elsewhere about the acceptability of this response rate for this type of survey [1]. For example, in Australia and elsewhere, survey response rates have been declining over the past decade as people become more active in protecting their privacy and more research is undertaken with the public (both market research and more ‘formal’ research) [80,81]. In the absence of a higher response rate, we have documented our efforts to increase the response rate [82]. As noted earlier, reminders postcards were sent to non-responders to ensure as high a response rate as possible [83]. Nevertheless, the potential for survey non-response bias is acknowledged for the Australian data and the lack of data on response rates is acknowledged for data collected in the other countries. 

The third potential limitation relates to the slight differences in the question wording and response category formats due to necessary language and cultural reasons. However, whilst still a limitation, Rice et al. identify that the design of international surveys needs to be mindful of the requirement for the cross-cultural equivalence of instruments [84]. We were cognisant from the survey design stage in terms of trying to make each country-specific survey as comparable as possible. In order to address this, we consulted with academics who comprise the Asia-Pacific Scientific Steering Group On Social Quality (Prof Ward is a foundation member of this group). It was decided that the wording for one independent variable, subjective health satisfaction, differed between the Australian survey and all other country surveys; Australia’s item options were ‘happy’, ‘average’, and ‘unhappy’ and all other countries’ were ‘satisfied’, ‘neither satisfied nor dissatisfied’, and ‘unsatisfied’. In addition, in Japan, it was decided not to include questions on political activities for cultural and political reasons.

A further potential limitation refers to cultural interpretation of survey questions. In order to minimise this, all surveys were piloted within each country before the full surveys were administered and any difficult questions were then discussed within the full Asia-Pacific research team and changed or deleted if necessary. Nevertheless, Robone et al. (2011) identify difficulties in using scale variables in cross-country comparisons because there is potential that when faced with the instrument individuals are likely to interpret the meaning of the available response categories in a way that systematically differs across populations or population subgroups [85] which compromises the comparability of the data in cross-country analyses. We recognise that by applying country level estimates within the analysis (mixed effects logistic regression models [86]), we can draw more insightful conclusions for comparing the data. However, this form of analysis was not possible given the slightly different predictor variables and different survey and sampling methods. Therefore, the aim of this manuscript is to report on *within* country results rather than cross country comparisons. Nevertheless, we do make tentative comparisons between countries, albeit not statistical, in order to highlight potential similarities and difference in SDH and where more research may be required. 

Furthermore, the amount of variance explained by the factors included in the regression models specified for all countries separately was generally small as indicated by low Nagelkerke R^2^ estimates. It is therefore acknowledged that the socio-demographic predictors included in the current investigation do not achieve substantial contributions to explaining response differences, hence further variables need to be investigated which might carry stronger predictive qualities for the outcome variables examined. Despite the comparatively small amount of variance explained, the models specified were highly significant which is taken as being indicative of small, yet significant contributions of socio-demographic variables to SDH. 

These potential limitations undoubtedly affect the extent to which the current findings may be generalised. Therefore, we acknowledge that limitations apply which mean the current results need to be interpreted and generalised with caution, and that replication is required to strengthen the existing body of evidence. Nevertheless, given the relative paucity of data in this area of interest, the current results add substantially to our current understanding of variables affecting SDH in different countries. 

The main empirical strengths of this paper are that we jointly developed, piloted and validated the survey tool for use in each of the six countries, undertook data collection at similar time periods and then we collated, input, cleaned and weighted the data using the same procedures in order to produce a single SPSS dataset. The original survey tool was developed and validated in English [25] and was then translated into relevant languages by the relevant academics within our Asia-Pacific research team [1,69–72]. Nevertheless, it is possible that some of the questions may have been difficult for respondents to answer (e.g. on experience of discrimination). The resultant representative samples for each country were large enough to enable multivariate logistic regression analysis. 

In terms of both conceptual and policy-related strengths, this paper presents the first attempt to conduct an analysis of cross-country social quality data with the specific purpose of identifying vulnerable population groups in terms of low socio-economic security, low social inclusion, low social cohesion and/or low social empowerment. Our analyses allow policy makers and researchers to identify population groups in need of policy and practice attention within countries. In particular, we have identified some population groups that have low levels of social quality across all four factors, identifying multiple and potentially cumulative disadvantages for these groups, discussed further in the next section.

### Summary of key findings

Table 24 provides a summary of the key findings from the regression models for each country. In Australia, low income groups had lower levels of all four social quality factors and people with poor subjective health had lower levels of all except socio-economic security. Therefore, any policy initiatives aimed at reducing poverty may be likely to impact positively across all four social quality domains. People with a chronic condition were more likely to experience discrimination, which adds weight to the findings on poor subjective health, providing added evidence that improving health (particularly for low income groups) may increase social quality. There were mixed findings in relation to age, whereby people aged less than 60 years had lower social cohesion and lower social inclusion whereas people aged 60 years or older had lower social empowerment. The findings related to younger people may be also related to being in the workforce, which may reduce opportunities for social cohesion and also increase potential for discrimination. These findings suggest the need for workplace policies to improve the social quality for these groups. In terms of specific population groups, males had lower levels of social cohesion, females had lower social inclusion, married/cohabiting people had lower socio-economic security and people undertaking household duties (mainly women) had lower social empowerment. 

**Table 24:** Summary of low levels of social quality across domains and countries.

	*Spent savings and borrowed money (low socioeconomic security)*	*Low level of membership (low social cohesion)*	*High levels of discrimination (low social inclusion)*	*Low participation in political actions (low social empowerment)*
*AU*	Low income Married/cohabiting	Low income Males <60 years Poor subjective health Paid employment	Low income Females <50 years Poor subjective health Chronic condition	Low income Household duties >60 years Poor subjective health
*HK*	Low income Married/cohabiting	Low income Married/cohabiting	Retired	>60 years Married/cohabiting
*JPN*	Low income Poor subjective health	No model	20-29 years Poor subjective health Part time employment	Question not asked in Japanese survey
*SK*	Low income Poor subjective health	Low income Paid employment Household duties	Low income Poor subjective health 20-29 years Chronic condition	Females >60 years
*THL*	Married/cohabiting Chronic condition Unpaid or unemployed	Low income Household duties	No model	Low income Good perceived health >50 years
*TWN*	Low income Unpaid or unemployed	Low income Unpaid or unemployed Part time employed	Low income 50-59 years	Low income Not married

In Hong Kong, people on low incomes had lower socio-economic security and lower social cohesion, and married people also experienced these in addition to lower social empowerment. Therefore, being married and on low income is particularly negative in terms of social quality in Hong Kong. Age is also important, since people 60 years or older had lower social empowerment and retired people had lower social inclusion. Therefore, older people in Hong Kong are less likely to be politically active and more likely to be discriminated against, potentially raising the opportunity for policy action geared towards increasing political activity and reducing age-based discrimination.

In Japan, no model was generated for social inclusion, and the question about political activity was not asked in the survey. For the remaining two social quality domains, people on low income had lower socio-economic security, people with poor subjective health had lower socio-economic security and lower social inclusion and people aged 20-29 years and who were part-time employed had lower social inclusion. Discrimination and socio-economic insecurity is therefore higher for younger and more marginalised groups (poor health, low income and part-time workers) in Japan.

In South Korea, people on low incomes and people with poor subjective health had both lower socio-economic security and lower social inclusion and people on low incomes additionally had lower social cohesion. These findings are similar to Australia, suggesting that similar policy initiatives may be developed, although these would need to be assessed for cultural suitability. Age was also an important factor, with 20-29 year olds experiencing lower social inclusion and respondents aged 60 years or older experiencing lower social empowerment, revealing more discrimination against younger people and less political activity amongst older people. Employment status was an important predictor of social cohesion, with people in paid employment and undertaking household duties both experiencing lower membership of organisations. This lower membership may be a result of people simply have less free time due to their employment or household duties, therefore limiting the opportunities for policy responses, unless those can be met within the workplace or home environments.

In Thailand, no model was generated for social inclusion. People on low incomes had lower social cohesion and lower social empowerment. Social cohesion was also lower for people undertaking household duties, a finding shared with South Korea. Married people and those on unpaid employment had lower socio-economic security, with the finding on marriage mirroring those of Australia and Hong Kong. In addition, people aged 50 years and older had lower social empowerment, a finding also in Australia, Hong Kong and South Korea, suggesting the potential for policies geared towards elders to increase opportunities for political activities and engagement in civic society.

In Taiwan, the findings in relation to low income groups were the same as Australia – people on low incomes experienced lower socio-economic security, social cohesion, social inclusion and social empowerment, revealing another example of multiple and possibly cumulative disadvantage for people on low incomes. Additionally, people in unpaid employment had lower socio-economic security and social cohesion and people in part-time employment also had lower social cohesion. These findings may be similar to the findings on low income, suggesting broader economic disadvantages in Taiwan. People aged 50-59 years had lower social inclusion and unmarried people had lower social empowerment. However, the main picture in Taiwan seems to be related to economic disadvantage as opposed to other socio-demographic factors.

In terms of socio-economic security, there were three key socio-demographic predictors. People on low incomes, in unpaid jobs or who were unemployed had lower levels of socio-economic security in all six study countries, revealing the pervasive influence of relative poverty on this domain of social quality. People with poor subjective health or a chronic condition had lower socio-economic security in Japan, South Korea and Thailand and married or cohabiting people had lower socio-economic security in Australia and Hong Kong. 

In terms of social cohesion, five countries had regression models and in those models, two key socio-demographic predictors emerged. Similar to findings for socio-economic security, people on low incomes had lower levels of social cohesion, showing that relative poverty impacts both socio-economic security and social cohesion in all countries. People reporting household duties had lower levels of social cohesion in South Korea, Thailand and Taiwan, which may be a reflection of socio-economic position but may also be gender related since females were predominantly the respondents nominating household duties as their employment status. From our data, we cannot provide a sociological explanation of the data, but simply present the regression models, leaving a space open for further research to understand the reasons for the statistical relationships.

For social inclusion, there were three key predictors across the five countries that had regression models, namely low income, age and poor health. People on low incomes had lower social inclusion in Australia, South Korea and Taiwan, which adds evidence to the critically important role of relative poverty in predicting low social quality. People with poor subjective health or a chronic condition had lower social inclusion in Australia, Japan and South Korea, which adds weight to the importance of poor health in shaping social quality since it was also a predictor of socio-economic security. Indeed, poor health was a predictor of both socio-economic security and social inclusion in South Korea and Japan, potentially suggesting the need for policy initiatives to improve social quality for people with poor health in these countries. Age was also an important predictor in all countries with a regression model, although for different age groups. Older people experienced lower social inclusion in Taiwan (50-59 years) and Hong Kong (retired), younger people in Japan and South Korea (20-29 years in both countries) and younger and middle-aged people in Australia. These data show the importance of age-related discrimination in all study countries which, given the different age groups experiencing discrimination, may require specific policies depending on whether older or younger people experience the discrimination.

In terms of social empowerment, both low income and age were strong predictors across a number of countries, although regression models were generated in only five countries. People on low incomes had lower social empowerment in Australia, Thailand and Taiwan. This finding shows that low incomes predict all of the four domains of social quality in both Australia and Taiwan, 3/3 domains in Thailand and 3/4 domains in South Korea, 2/4 domains in Hong Kong and 1/2 domains in Japan. Older age was also an important predictor of social empowerment, with people aged 60 years or older having lower social empowerment in Australia, Hong Kong and South Korea, and people aged over 50 years having lower social empowerment in Thailand. In South Korea and Australia, younger people experience lower social inclusion and older people experience lower social empowerment, whereas in Hong Kong, older people experience both lower social inclusion and lower social empowerment. These data may suggest the need for particular older-age policy in Hong Kong to decrease discrimination and increase political participation. 

## Conclusions

It is recognised globally that public health policy, practice and research needs to focus on addressing the SDH in order to increase the health of the most vulnerable and disadvantaged groups [3,34,35,87,88]. Given the multiple and complex nature of the SDH, this paper used a new conceptual framework called social quality, which we argue allows researchers and policy makers to measure and respond to the SDH. In a previous paper, we argued for the utility of social quality for researching the SDH [1], and in this paper, we provided empirical evidence of the socio-demographic predictors across six countries in the Asia-Pacific region. Our analyses focused on the four domains of social quality: socio-economic security, social cohesion, social inclusion and social empowerment, which we argue are key SDH. As such, our paper represents a key social epidemiological analysis of the SDH, and in particular, an important contribution to identifying vulnerable populations groups in need of policy and practice responses in the Asia-Pacific region.

Overall, our results also provide baseline measures for identifying where and how policy could be altered to improve social quality and therefore, the SDH. Furthermore, these data may be used for future policy evaluation to identify whether changes in policy have indeed improved social quality and the SDH, particularly for marginalised and vulnerable populations. 
